# Hierarchical Action Control: Adaptive Collaboration Between Actions and Habits

**DOI:** 10.3389/fpsyg.2019.02735

**Published:** 2019-12-11

**Authors:** Bernard W. Balleine, Amir Dezfouli

**Affiliations:** ^1^Decision Neuroscience Laboratory, School of Psychology, University of New South Wales Sydney, Sydney, NSW, Australia; ^2^Data 61, Commonwealth Scientific and Industrial Research Organisation, Sydney, NSW, Australia

**Keywords:** goal-directed action, habits, action sequences, chunking, model-based, model-free, reinforcement learning

## Abstract

It is now commonly accepted that instrumental actions can reflect goal-directed control; i.e., they can show sensitivity to changes in the relationship to and the value of their consequences. With overtraining, stress, neurodegeneration, psychiatric conditions, or after exposure to various drugs of abuse, goal-directed control declines and instrumental actions are performed independently of their consequences. Although this latter insensitivity has been argued to reflect the development of habitual control, the lack of a positive definition of habits has rendered this conclusion controversial. Here we consider various alternative definitions of habit, including recent suggestions they reflect chunked action sequences, to derive criteria with which to categorize responses as habitual. We consider various theories regarding the interaction between goal-directed and habitual controllers and propose a collaborative model based on their hierarchical integration. We argue that this model is consistent with the available data, can be instantiated both at an associative level and computationally and generates interesting predictions regarding the influence of this collaborative integration on behavior.

## Introduction

Although it has long been debated how precisely actions variously called volitional, voluntary or goal-directed should be defined, over the last 20 years or so it has proven fruitful to define as goal-directed those actions demonstrably sensitive to changes in: (1) the causal relationship to their consequences and (2) the value of those consequences (Balleine and Dickinson, 1998). When the performance of an action demonstrates sensitivity to both of these changes, it is defined as goal-directed; when its performance is insensitive to these changes, it is not. By taking this approach, considerable progress has been made not only in providing evidence for goal-directed action in a variety of species (including humans!) but also for the neural bases of these kinds of action. In addition, the usefulness of these tests to delineate goal-directed from non-goal-directed actions has inspired various investigators to apply them as a means of establishing whether the performance of an action reflects the operation of a second form of action control, usually referred to as habits.

Despite their apparent simplicity, habits are actually quite complicated. Although most theories of habit are very clear about what they are – referring to their non-cognitive, repetitive regularity, their stimulus control, and so on – demonstrating that an action is a habit is not straightforward. For example, numerous papers have advanced the idea that a habit is an action that is *insensitive* to changes in the action-outcome relationship and in outcome value (reviewed in Balleine and O’Doherty, 2010). However, in practice, where the effects of such changes are evaluated against some control group, this has meant asserting the null hypothesis. Thus, for example, when the experimental group differs from, say, a non-devalued or a non-degraded control, then performance of the former is regarded as goal-directed. However, when these experimental and control groups do not differ, then performance of the former is regarded as habitual. Furthermore, these criteria fail to differentiate habits from other forms of reflex; for example, although habits are insensitive to changes in the action-outcome relationship, so are Pavlovian conditioned reflexes (although, whereas habits are insensitive to devaluation, Pavlovian CR’s often are not; cf., Dickinson and Balleine, 1994, 2002). The general problem, however, is asserting an action is a habit when it fails to satisfy the tests for goal-directed action, because this does not discriminate that action from performance when the actor is simply confused, forgetful, or having trouble integrating beliefs regarding action outcomes with their desire for a particular outcome. In such cases, behavior may appear habitual when it is in fact controlled by a faulty goal-directed controller. This could be the case in people suffering psychiatric conditions, addictions, or brain damage of various kinds and in such cases although the evidence might confirm their behavior is not normatively goal-directed, it may not be entirely habitual either.

## Competition Between Goal-Directed and Habitual Control

**Figure 1:**
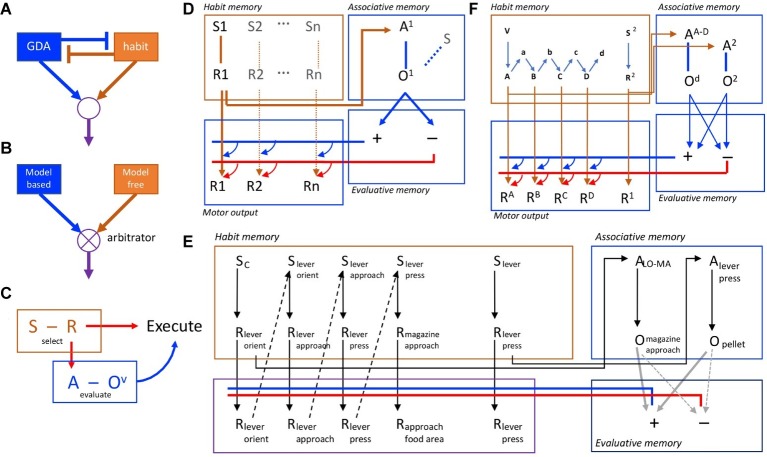
Competition and collaboration in goal-directed and habitual action control. **(A)** Simple model of competition for performance with goal-directed and habitual controllers mutually inhibiting one another. **(B)** More sophisticated approach to competition, with goal-directed and habitual controllers competing through arbitration. **(C)** Behavioral evidence suggests, in contrast to competition, that habit and goal-directed processes are intimately connected and collaborate in action selection, evaluation, and execution. **(D)** A formal associative architecture that instantiates the collaboration between habit and goal-directed controllers through the interaction of habit memory and associative memory systems, the latter feeding back to control performance. Action selection in the habit memory is mediated by the association of S1 and R1 that feeds forward to provide both subthreshold activation of the motor output and activation of the action representation, A1, in the associative memory provoking retrieve of the action outcome (O1) and its evaluation through the interaction of the associative and evaluative memory systems. The latter provides a promiscuous, feedback (cybernetic) signal that sums with the forward excitation from the habit memory. If positively evaluated (blue lines/arrows), it provokes action execution; if negatively evaluated (red lines/arrows), it blocks performance. **(E)** An example of the representation of a complex habit sequence in the habit memory incorporating lever press and magazine approach responses together with a simple lever press action. Both are represented in the habit memory (the expanded sequence, the acquisition of which is supported by proprioceptive feedback from motor output) and its chunked representation in the associative memory (e.g., A^LO-MA^). **(F)** The formal associative-cybernetic model incorporating chunked action sequences and simple actions in both the habit memory and the associative memory.

This latter criticism has obvious implications for how habits should be defined but also affects how we should think about the way that habitual and goal-directed actions interact. Generally speaking, the consensus supposes these forms of action control as competing, at least as far as instrumental performance is concerned (Figure 1A). At a behavioral level, for example, it is usual to point, first, to the relatively clear evidence that distinct associative processes underlie the two forms of action control; whereas goal-directed actions depend on the action-outcome association, habits are commonly thought to involve a process of stimulus-response association (Dickinson, 1994). Based on this distinction, various dual process accounts of the way these distinct learning processes influence instrumental performance have been developed, perhaps the most influential of which suggests that, whereas an action, such as lever pressing in rodents, begins under goal-directed control, the net influence of the action-outcome association declines as the strength of the S-R association increases until the influence of the latter exceeds the former and so takes over motor control (Dickinson et al., 1983; Dickinson, 1985, 1994). And, indeed, a number of studies have reported behavioral evidence consistent with the dual process perspective (reviewed in Dickinson and Balleine, 2002).

### Neural Evidence

Considerable evidence for competition has also come from studies assessing the neural bases of these two forms of control. Thus, for example, sometime ago we reported evidence that lesions of the prelimbic prefrontal cortex, the dorsomedial striatum, and the mediodorsal thalamus had in common the effect of reducing the sensitivity of instrumental performance to changes in both the action-outcome relationship and outcome value; i.e., compromising goal-directed control appeared to cause a reversion to habit, consistent with the idea that these control processes compete (Balleine, 2005; Balleine et al., 2007; Balleine and O’Doherty, 2010). Following the criticism above, however, loss of sensitivity to tests of goal-directed action may not necessarily mean the action has become a habit and may instead reflect a loss in the accuracy of retrieval or in translating learning to performance. Again, what is required to support this claim is positive evidence that a loss of goal-directed control increases habitual control.

There are two other sources of positive evidence from studies assessing the neural bases of action control consistent with a competitive process. The first comes from findings suggesting that one effect of goal-directed control is to inhibit the performance of habits (Norman and Shallice, 1986). For example, although extensively trained instrumental actions are insensitive to outcome devaluation, this insensitivity is only observed in tests conducted in extinction; i.e., in a situation in which outcome delivery is withheld and so does not provide direct and immediate negative feedback. When feedback is provided, by delivering the devalued outcome contingent on the action, then the performance of even extensively trained actions rapidly adjusts; punishment appears to result in response suppression which is as rapid for an extensively trained action as a relatively modestly trained one (see, for example: Adams, 1982; Dickinson et al., 1983, 1995). Importantly, damage to, or inactivation of, the neural network mediating goal-directed control attenuates this effect of punishment and results in the persistence of an action even when it delivers a demonstrably devalued outcome (one, for example, that the animal will not consume; e.g., Balleine et al., 2003; Yin et al., 2005). Second, a number of studies have found that damage to, or inactivation of, the dorsolateral striatum (Yin et al., 2004), or structures interacting with dorsolateral striatum, such as the central nucleus of the amygdala (Lingawi and Balleine, 2012), can block habitual control resulting in even extensively trained actions remaining goal-directed. Although also consistent with other accounts (see below), this effect is nevertheless consistent with a competitive interaction between habitual and goal-directed control processes and the view that, at least in some situations, when habits are inhibited goal-directed action control is liberated from its competing influence.

Nevertheless, other features of habitual performance tend, on their face, to reduce the importance of much of this evidence for competition between control processes. One factor is the increase in the speed of performance commonly observed to accompany habits. For example, a number of studies have found evidence that the speed of both response initiation (reaction time) and motor movement is increased with experience. Thus, biases in reaction time appear to depend on parameters experienced during prior training rather than new computations (Wong et al., 2017) and are manifest as costs when conflicting response strategies, involving the repetition of movements at a particular speed or toward a particular direction, influence the kinematics of subsequently performance (Huang et al., 2011; Verstynen and Sabes, 2011; Hammerbeck et al., 2014). This is true of studies of human action but has also long been claimed in studies of habit in animals, especially rodents working in runways of various kinds (reviewed in Bolles, 1967, ch. 8). The suggestion that performance speed increases as actions become habitual in some ways trivializes competition between goal-directed and habitual controllers because, if habits are faster, they could potentially be completed before the goal-directed system is engaged and so will not directly compete with goal-directed control. Indeed, it is this speed of action that allows us to make sense of the errors that habits bring; e.g., the planning errors and slips of action apparent in selecting or completing an action that will otherwise result in an unwanted, devalued, or aversive outcome. It can also explain the reversion to goal-directed action induced by inactivation of the dorsolateral striatum; when habitual control is offline, there is simply more time to implement goal-directed control.

### Computational Evidence

But what of evidence that goal-directed control inhibits habits? Another class of account consistent with a competitive view has been driven by the computational descriptions of goal-directed and habitual action control derived from distinct forms of reinforcement learning (RL): model-based RL in the case of goal-directed action and model-free RL in the case of habit (see Dolan and Dayan, 2013 for review). The former views goal-directed control as a planning process; the actor foresees the future actions and the transitions between future states necessary to maximize reward *via* a form of tree search and integrates these into an internal model of the environment. In contrast, model-free RL supposes that action selection in a particular state is determined by the predicted long run future reward value of the action options in that state. Within this literature, whether an agent selects goal-directed or habitual control has been argued to be the outcome of a competitive arbitration process (Figure 1B); computationally, the actor selects the control process for which the state-action value is least uncertain (Daw et al., 2005). And this is true too of more recent accounts; whether framed in terms of reliability (Lee et al., 2014) or costs and benefits to determine the outcome of arbitration (Pezzulo et al., 2013; Shenhav et al., 2013; Keramati et al., 2016), they also contend that actions and habits compete for control. Treatments (whether behavioral or neural) that influence arbitration will be predicted to influence the balance between goal-directed and habitual control; viz., if reduced reaction times bias arbitration toward a model-free process, then perhaps the delivery of an unexpected aversive or noxious outcome biases arbitration toward a model-based one.

Evidence for competitive model-based and model-free controllers has been most clearly derived from computational analyses of performance on a class of task that attempts to pit goal-directed and habitual choices against one another in a multistage discrimination situation (Daw et al., 2011). The aim of this task is essentially to set up a continuous revaluation procedure across trials. In one version, a first stage choice transitions probabilistically to one of two second stage states, one with a higher probability than the other. At the second stage, a choice results, again probabilistically, in reward or no reward, the probability of which changes slowly throughout the task to encourage the decision-maker to sample new options. The task is, therefore, structured on a RL view of the world with explicit states and action-related transitions between those states. Importantly, it is assumed that learning that a choice in the second stage state results in reward (or no reward) revalues (or devalues) that state as a goal. The question then becomes: does the decision-maker take advantage of that information or not? If so then their choice is *assumed* to reflect planning based on the interaction of the stage 1 and stage 2 states and so to be model-based (or goal-directed). If not, it is *assumed* that the choice merely recapitulates prior performance and is model-free (or habitual).

Based on these assumptions, the stage 1 choices of human subjects on the two-stage task show a mixture of model-based and model-free control (Daw et al., 2011) that can be biased toward one or other process by a variety of factors; e.g., amount of training (Gillan et al., 2015), cognitive load (Otto et al., 2013), altered activity in the dorsolateral frontal cortex (Smittenaar et al., 2013), and (likely relatedly) *via* the influence of various psychiatric conditions (Gillan et al., 2016). Although this intermixing of controllers is consistent with variations in the influence of competitive controllers, trial-to-trial variation presents something of a puzzle, the explanation of which – if we are to maintain this perspective – returns us to the issue of arbitration.

Importantly, while there are computational theories of arbitration (e.g., Griffiths et al., 2015), whether an arbitrator actually regulates the contribution of each system remains unknown. And, in fact, analyses that break the world into discrete states may not be the best way to assess this problem. Although such analyses may be helpful both when the experimenter is trying to tie neural events to behavioral responses or is hoping, computationally, to apply a reinforcement learning approach to these tasks, the original data that inspired our understanding of distinct goal-directed and habitual forms of action control came from continuous, self-paced, unsignaled situations in which humans and other animals explore the environment, discover its structure, learn new actions and their causal consequences, and then utilize that knowledge to maximize reward. Non-human animals in particular encode these relationships based on their own experience and not *via* the instructions of the experimenter. In contrast, what human participants learn on multistage discrimination tasks can be difficult to discern and may not accord with the assumptions of model-based and model-free RL analyses as to the drivers of performance. There are issues in establishing whether the assumptions from model-based and model-free reinforcement learning are consistent with the subjects’ behavior; how accurately they update common and rare transition probabilities; how large the state-space that subjects use to make choices actually is (see Akam et al., 2015 for discussion). Furthermore, other factors, such as performance rules or environmental cues, including the stimulus predictions embedded in the task, could also influence performance; indeed, it has never been clear why experimenters commonly use both actions and stimuli to predict the second stage states in two-stage tasks. Another factor recently suggested to influence arbitration between model-based and model-free control is the integration of the costs and benefits of each system; i.e., the rewards based on the average return of model-based and model-free control against which are contrasted the intrinsic cost of model-based control (Kool et al., 2016). Interestingly, evidence has been collected from novel versions of the two-stage task suggesting variations in reward value and costs based on planning complexity can alter the model-based and model-free trade-off (Kool et al., 2017, 2018). Importantly, however, these factors do not appear to influence arbitration on the original version of the two-stage task, likely due to its intransigence in the calculation of reward estimates due to a lack of access to the second stage reward outcomes (Kool et al., 2016). Indeed, whereas model-based and model-free RL provide reasonable simulations of the first stage choices of the two-stage task, experimenters investigating these positions have typically not generated predictions about what animals will do on the second stage choice (Dezfouli and Balleine, 2013, 2019). It is clear, therefore, that our understanding of what animals and humans are actually doing on these complex tasks is very far from settled.

Taken together, these issues concerning the behavioral, neural, and computational evidence for competition between action controllers raise significant questions regarding: (1) how habits are best characterized; (2) the kind of evidence that we should accept for their occurrence; and (3) whether explaining their interaction with goal-directed control requires the generation of a third kind of quasi-controller positioned to arbitrate between the other two. Fortunately, there are other accounts available that allow us to move beyond each of these issues.

## Collaboration Between Controllers

Against the competition view, alternative positions have been developed proposing that goal-directed and habitual controllers collaborate to coordinate instrumental performance. In the past, we have described a number of sources of behavioral evidence for this perspective (Balleine and Ostlund, 2007), among the strongest of which comes from studies assessing the factors controlling the selective reinstatement of instrumental actions (Ostlund and Balleine, 2007). The basic phenomenon was established as an assessment of the effects of outcome delivery on subsequent action selection. Rats trained on two actions for distinct outcomes were then given a period of extinction on both actions until performance was completely withheld. At that point, one or other of the two outcomes was delivered non-contingently. The question at issue was what the free outcome delivery would produce; if the outcome retrieved the action with which it was associated then we should expect that action to be selected and executed, and that is what we observed. Subsequently, we sought to assess whether the outcome selected the action that delivered the non-contingent outcome as a goal or whether that outcome served as a stimulus that retrieved the next performed action. To achieve this, rats were again trained on two actions for different outcomes; however, each action-outcome pair was trained in alternation; i.e., A1 → O1 was always followed by A2 → O2. Again, both actions were extinguished before we assessed the effects of non-contingent outcome delivery on the reinstatement of A1 and A2. If an outcome retrieves the action that delivered it as a goal, then delivering, say, O1 should retrieve A1. If, however, O1 acts as a stimulus that retrieves the next action, then O1 should retrieve A2. In fact, we found the latter result; outcome-specific reinstatement appears to reflect the effect of a forward outcome-response association on performance. Furthermore, this effect was not diminished by devaluing the reinstating outcome suggesting that outcome-mediated response retrieval is not dependent on the outcome’s value but on its stimulus properties. This result suggests, therefore, that instrumental action *selection* is initiated by a form of S-R process in which the stimulus properties of the outcome are the proximate cause of action retrieval (Balleine and Ostlund, 2007).

Importantly, subsequent studies found that, when retrieved in this way, it is the outcome that serves the selected action as a goal that mediates the *execution* of the action. To establish this, we used a similar training situation except that the outcomes were used as explicit discriminative cues for action selection, and found that these kinds of stimuli can, in fact, engage an evaluative process but of the action subsequently retrieved by those discriminanda (Ostlund and Balleine, 2007). Devaluing the outcome that served as a goal for the retrieved action reduced the vigor of performance but not the ability of the outcome to serve a discriminative cue, consistent with other reports using more traditional discriminative stimuli (Colwill and Rescorla, 1990; Rescorla, 1994). That is, performance, but not action selection, was attenuated if the outcome earned by the reinstated action was devalued. In the ordinary course of events, therefore, the outcome controls actions in two ways: (1) through a form of S-R, or ideomotor, association in which the stimulus properties of the outcome can select the action with which they are associated; and (2) through the standard R-O association in which a selected action retrieves its specific outcome as a goal. Clearly, the subsequent retrieval of the value of the outcome is a necessary step toward the actual performance of the action. Hence, this behavioral evidence suggests that a selection-evaluation-execution sequence lies at the heart of instrumental performance and that this control requires the collaborative integration of habitual S-R and goal-directed R-O control processes (Figure 1C).

### Cybernetic Control

At least two kinds of account accord with this collaborative control process. The first, advanced some years ago, is what has become known as the associative-cybernetic model of instrumental performance (Dickinson and Balleine, 1993). This account has its origins in Thorndike’s (1931) ideational theory of instrumental action proposing that a stimulus that evokes a response urge or tendency calls to mind the consequences of the action selected by that tendency and these two processes – driven essentially by stimulus–response and action-outcome associations – check or favor one another to release action execution. In addition to providing a clear basis for the collaborative integration of habitual and goal-directed controllers, this view also has the merit of providing an answer to one of the thornier questions; why do we do anything at all? Early cognitive theorists, concerned by the poverty of the stimulus-response approach, developed models of action based on more elaborate internal variables (e.g., Tolman, 1932). Nevertheless, how thought initiates action remained an ongoing issue; the concern being, as Guthrie put it, that such views left the actor buried in thought (Guthrie, 1935). When and why does thinking about actions and their consequences stop and acting begin? Thorndike’s account suggests that it is external stimuli rather than thoughts that initiate this process by urging a response; that the action and its consequences are brought to mind only subsequently, at which point the value of the latter provides the basis for either checking the urge, when the consequences are punishing, or favoring it, when they are rewarding, thereby providing the necessary feedback to modulate action execution.

These ideas have been developed in a number of ways to capture both the behavioral data on instrumental performance and their neural bases (reviewed elsewhere; Dickinson and Balleine, 1993; Dickinson, 1994; Balleine and Ostlund, 2007). Generally, it has been suggested that a stimulus–response memory interacts with an associative memory to drive the retrieval of a specific action and its consequences, that the latter retrieves an incentive memory of the outcome that, by marshaling specific motivational and emotional processes, determines the value of the outcome, to potentiate or de-potentiate the motor signal associated with the response tendency of the S-R memory, thereby increasing the probability that the action will be executed. It is this latter process that constitutes the cybernetic or feedback component of the model (Figure 1D).

### Hierarchical Control

Alternatively, we have recently argued that goal-directed and habitual control processes interact in a hierarchical manner; i.e., that habits are selected by a goal-directed control process as one means of achieving a specific goal (Dezfouli and Balleine, 2012). Within this account, although habits are often described as single-step actions, their tendency to combine or chunk with other actions and their insensitivity to changes in the value of, and causal relationship to, their consequences suggest that they are better viewed as forming the elements of chunked action sequences. In this context, chunking means that the decision-maker treats the whole sequence of actions as a single action unit and so the individual actions of which the sequence is composed are represented independently of their individual outcomes. As a consequence, the value of an action sequence will be established independently of the individual action-outcome contingencies and the values of the outcomes of the action elements inside the sequence boundaries, which will be invisible to the decision-maker. Once selected, each action will then be executed in the order determined by the sequence in an open loop manner; i.e., without further feedback from their individual consequences.

### Integrating Cybernetic and Hierarchical Control

In fact, hierarchical and cybernetic control are not mutually exclusive and, indeed, starting with James (1890), there has been a long tradition of associative accounts of action sequences, particularly from within the behaviorist tradition that used stimulus-response sequences to explain apparently cognitive control processes. A good example of this approach is Hull’s explanation of latent learning. Tolman, for example, was able to demonstrate that changing the value of a specific goal box in a previously explored maze by giving a rat food in that box was sufficient immediately to alter the speed and accuracy with which the rat reached the goal subsequently without the need for additional training (Tolman and Honzik, 1930). The natural interpretation of this effect is that the rat had learned about the change in value of the goal and was able to incorporate that knowledge into what it knew about the structure of the maze to alter its choice performance, much as we have argued for goal-directed actions generally. In response to effects like this, however, behavioral theorists introduced the fractional goal-response, responses such as chewing or licking, that, when associated with other responses within the maze, could form a sequence able to explain choice performance without resorting to goal-directed control (Hull, 1952).

Although these kinds of explanation are no longer favored for goal-directed actions, they give a feeling for how an account of habits in terms of action sequences might be constructed and deployed. In the simplest case, it would apply to overtraining-induced habits by arguing that the target action, say lever pressing, is incorporated into a sequence with other common responses performed around the lever press response; e.g., lever orienting, lever approach, lever press, magazine approach, magazine entry, magazine exit, lever orient, and so on (see Figure 1E). Initially, these sequences of responses would be purely incidental; the simple component action of lever pressing is sufficient and any tendency to press the lever will call to mind the action-outcome relationship resulting in outcome evaluation and the execution or suppression of the action. With practice, however, chunking these component responses together would allow the whole sequence to run off rapidly and smoothly using minimal cognitive resources. There are, however, costs associated with this form of action control; chunking these component responses together may allow stimuli antecedent to the response tendency to set off the habitual chain without requiring the animal to monitor each component action, however it will also render the consequences of responses within the chain and the value of those consequences invisible to the decision maker. If such sequences are structured and selected independently of their simpler component actions, such as lever pressing, and if the sequence’s relationship to and the value of its outcome are not dependent on these component actions, then one can immediately see how, when chunked within a sequence, a target action can appear insensitive to changes in its relationship to and the value of its programmed consequences (cf. Dezfouli and Balleine, 2012; Dezfouli et al., 2014).

Within the associative-cybernetic model, habit sequences would form within the habit memory through the integration of responses, perhaps *via* their feedback; i.e., the proprioceptive stimuli they evoke. This response-response chaining is what is meant by the chunking of an action sequence and, as an action, it can be selected in the associative memory just as any other action is selected; i.e., a response tendency, initiated in habit memory, activates the action sequence representation and its outcome in associative memory. If positively evaluated, each subsequent response will be executed without evaluation until the sequence is terminated (see Figure 1F).

Although it was argued above that such an account can explain why habits are insensitive to degradation and devaluation treatments, it might be asked, if the outcome of the sequence needs to be evaluated positively for the sequence to be initiated, why devaluation does not result in a reduction in the production of the overall sequence. The answer to this is that it can do so if the outcome that is devalued is the outcome associated with the sequence (Ostlund et al., 2009). If, however, the outcome that is devalued is associated with a response *inside the sequence boundaries,* then the devalued outcome will be invisible to the associative memory and will not be evaluated. In this case, the sequence will persist despite devaluation. That something like this must be going on is suggested by the fact that, after overtraining, habitual lever presses in rats have been found to become more sensitive to devaluation over the course of extinction as, presumably, the press-approach sequence described above was broken down (Dezfouli et al., 2014).

More direct evidence for this account has recently been reported by Ostlund and colleagues (Halbout et al., 2019). In this study, rats were trained to lever press for a food pellet reward before the goal-directed nature of this response was assessed using an outcome devaluation assessment conducted in extinction. The investigators developed a novel microstructural analysis of the performance of the animals during training and test, investigating the tendency to press the lever but also the degree to which such presses were followed by approach responses to the food magazine and how the relative incidence of these responses changed after devaluation. Importantly, they found evidence that the rats used two different strategies when initiating the lever press response, performing it as part of an action chunk (press-approach) or as a discrete action (press only). Consistent with an account in terms of habitual sequences, these distinct strategies appeared to be differentially sensitive to reward devaluation; whereas the rats were generally less likely to lever press for the devalued than for the valued reward, the press-approach chunk was found to be less sensitive to reward devaluation than presses that were not followed by approach. Furthermore, the proportion of chunked lever press-approach actions was actually greater for the devalued action than for the valued action. This suggests there was a change in the willingness to select the chunked sequence on the devalued relative to the non-devalued action, consistent with the claim that the sequence had a higher value than the individual lever press after devaluation.

Generally, therefore, we argue that hierarchical control can be accommodated within an associative-cybernetic account of instrumental conditioning. In fact, it appears to be well suited to this account with individual actions and chunked action sequences sitting at the same level in the associative memory and with simple or serially chained stimulus–response associations sitting at the same level in habit memory. This account is also consistent with several other features of habitual control. First it is consistent with the increased speed of habit execution: without having to evaluate the individual actions through the cybernetic feedback component of the model, the action sequence can run off more rapidly than if each response is evaluated. Second, this account addresses slips of actions by pointing to the chaining of responses at a mechanistic level. Appropriate response feedback will initiate the next action in a chain irrespective of the outcome of that response (Matsumoto et al., 1999). Furthermore, feedback relating to a response in the middle of a chain should be expected to result in a “capture error”; i.e., in the completion of that chain even when the animal is pursuing some other outcome (Norman and Shallice, 1986).

## Evidence for Hierarchically Organized Collaboration

Given that hierarchical control can be implemented within an associative-cybernetic architecture that requires the integration of goal-directed and habit controllers to explain instrumental performance, what evidence exists for this kind of collaboration? Here we describe two sources of evidence from human and rodent subjects consistent with this account, both taken from performance on the two-stage task described above.

### Human

As mentioned, the two-stage task developed by Daw et al. (2011) essentially arranges for changes in value to occur while the decision-maker is faced with an ongoing series of binary choices. Repeating past choices is assumed to be driven by the habit controller; altering choices in accord with predictions of future outcomes is assumed to be driven by the goal-directed controller. Critically for this analysis, all previous assessments of these factors have focused purely on stage 1 choices largely because popular reinforcement learning descriptions of choice on this task, i.e., model-based and model-free RL, only make differential predictions regarding stage 1 choices. However, it should be clear that, because the hierarchical-cybernetic model described above views habits as sequences of responses nested within a goal-directed controller and treats all actions as requiring collaboration between habit and goal-directed control, this approach is unique in making differential predictions not just for the first stage choices but also for second stage (and indeed for further) choices too.

**Figure 2:**
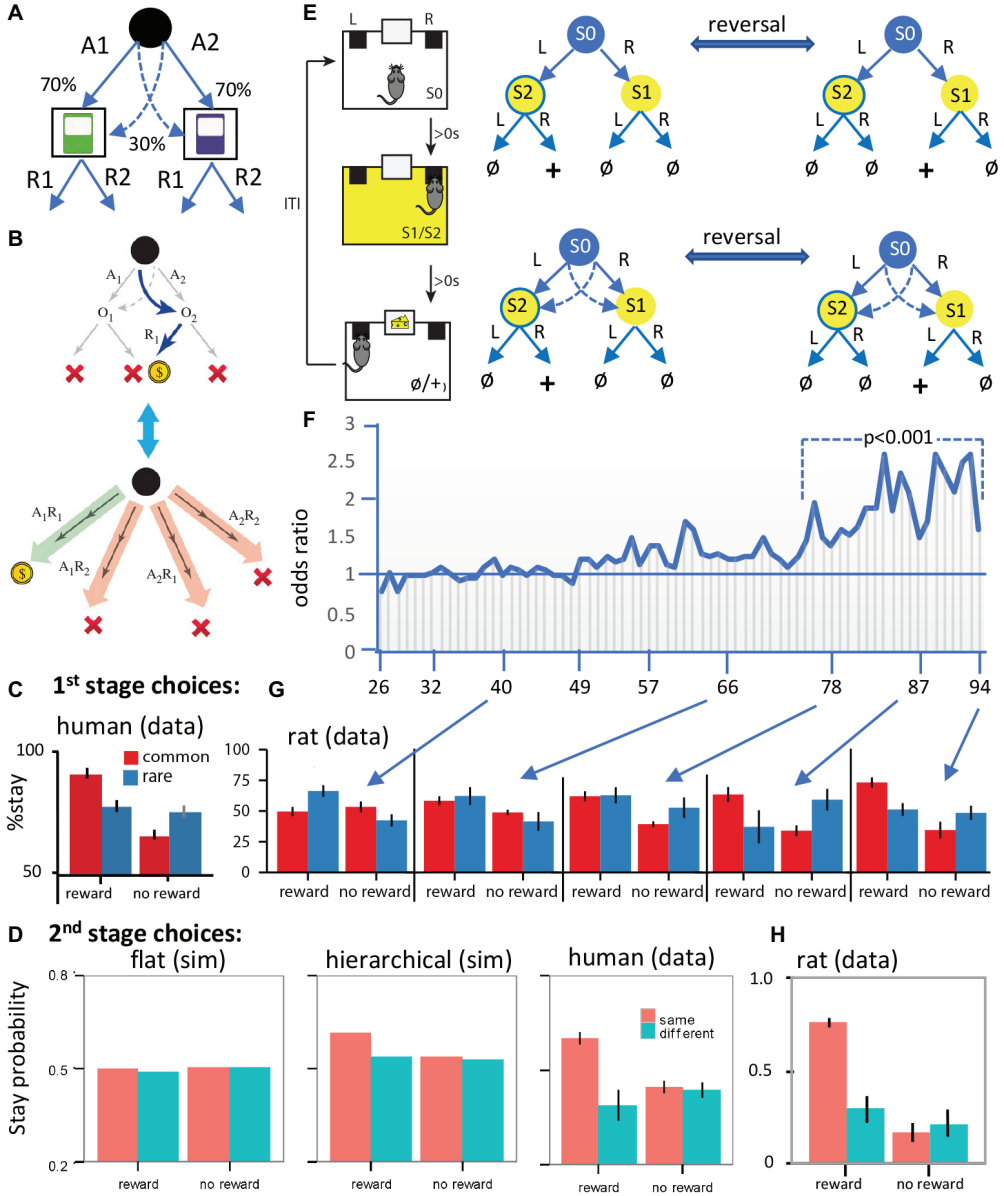
Evidence for hierarchical collaboration in humans and rats. **(A)** Two-stage task in human subjects. **(B)** After a rare transition (example shown) and revaluation of O2 (upper panel), an expanded action repertoire using action sequences (e.g., A1R1) can induce insensitivity to revaluation of the second stage choice (e.g., R1). **(C)** The influence of reward and non-reward on the tendency to stay on the same first stage choice after a common and a rare transition in human subjects. **(D)** Simulated (sim) second stage choices from various flat model-based and/or model-free RL models (left panel), a hierarchical RL model (center), and the human data (right panel). **(E)** Design of a two-stage task in rats with training conducted on a two-stage discrimination that is reversed, initially, every four trials and subsequently every eight trials. At various points in training, we included rare transitions as probe tests (sessions 40, 66, 78, 87, and 94). **(F)** The odds ratio of staying on the same stage 1 action after reward on the previous trial over the odds ratio after no reward. The horizontal line represents the indifference point. Each vertical line is one session. **(G)** Results from the probe tests. Note the comparable performance of rats and humans when rats show evidence of having acquired an accurate representation of the multistage nature of the task. **(H)** Rat data from second stage choices using a comparable version of the task to that used in humans. Panels **(A–D,G,H)** are taken directly from Dezfouli and Balleine (2013, 2019). Panels **(E,F)** are redrawn from Dezfouli and Balleine (2019).

We constructed a version of the two-stage task – see Figure 2A (cf. Dezfouli and Balleine, 2013 for details) – in which human subjects were instructed to make a binary choice at stage 1 (i.e., A1 or A2), the outcome of which was either O1 or O2, which were distinct two-armed slot machines. Subjects could then make a second binary choice in stage 2, choosing one or other arm (i.e., R1 or R2), and were then rewarded or not rewarded for their choice. We arranged the relationship between the stages as in previous reports of this task: i.e., A1 commonly led to O1 and A2 to O2; however, on rare trials, A1 led to O2, and A2 to O1. As a consequence of this arrangement, the role of stage 2 choices was, essentially, to manipulate the value of O1 and O2 and, in order to revalue the outcomes during the session, the probability of reward following each stage 2 choice increased or decreased randomly on each trial, causing frequent devaluation or revaluation of the O1 and O2 outcomes during the course of the task. Whereas changes in outcome value are usually accomplished by offline treatments, such as specific satiety and taste aversion learning, in this task values are changed through exposure to rare transitions inserted among the more common transitions.

Replicating previous reports, we found that stage 1 choices were sensitive to this form of revaluation, confirming that these actions were goal-directed – Figure 2C (human data). However, and more importantly, because two steps are required to reach reward it is possible for subjects to expand their choice options from A1 and A2 by combining stage 1 and stage 2 actions to construct action sequences; i.e., A1R1, A1R2, A2R1, A2R2 and to choose between these options based on their relationship to reward – see Figure 2B. Although the choice of stage 2 action (R1 vs. R2) should be based on the outcome of the stage 1 action, we found that, when the previous trial was rewarded and subjects repeated the same stage 1 action (A1 or A2), they also tended to repeat the same stage 2 action (R1 or R2), irrespective of the outcome of the stage 1 action. In these cases, the stage 2 action was determined at stage 1 when the sequence was executed. This observation of the open-loop execution of actions was not due to the generalization of action values from the common to the rare second stage outcome (e.g., using the example in Figure 2B; from O1 to O2). If this were the case, then subjects should have been more likely to repeat the same stage 2 action irrespective of the stage 1 action chosen. However, subjects had a higher tendency to repeat the same stage 2 action (e.g., O2) only when they executed the same stage 1 action (e.g., A1) – Figure 2D.

Recall that, according to the hierarchical approach, actions will be habitual if they fall within the boundaries of a sequence. And that is the case here; the outcomes of the stage 1 choices (i.e. O1 and O2) fall within the boundaries of the action sequences, consistent with the claim that these sequences were not always revalued during rare trials. This finding suggests that subjects should also make systematic errors after revaluation; i.e., if revaluation occurs on a rare transition then selecting the same action sequence performed on the previous trial means the subject must have ignored the fact that it was the alternative stage one action that was revalued. And indeed, consistent with this, reward on the previous trial increased the likelihood of repeating the same stage 1 action, whatever outcome and stage 1 action was revalued. Importantly, as previously reported, we also found performance to be a mixture of responses apparently insensitive to outcome revaluation and those sensitive to these manipulations. On previous accounts, such findings were argued to reflect competition between model-based and model-free controllers. On the hierarchical account, however, this merely reflects the difference between a model-based controller selecting simple actions (A1 and A2) on the one hand and habit sequences (A1R1, A1R2, A2R1, and A2R2) on the other. Importantly, we found evidence that, whereas model-based and model-free RL were as successful as a hierarchical RL model in simulating the stage 1 choices, only the hierarchical RL model could capture the stage 1 and stage 2 choices, and this superiority was established using Bayesian comparison between these different model families – see simulations in Figure 2D.

Generally, therefore, this study found evidence of action sequences that were insensitive to a change in outcome value, a finding that is uniquely addressed by the collaborative hierarchical account. Another feature of this account is that it provides a straightforward reason why the chronometry of action and habits should differ. Any attempt to evaluate each simple action before execution will necessarily slow the temporal dynamics of choice between the two stages compared to habit sequences, which can run off continually in open loop fashion without intervening evaluation. As such, when the second action in the sequence is not taken at stage 2, then reaction times should increase. We found evidence for this prediction in the data: if the previous trial was rewarded, reaction times were significantly faster (<379 ms) when a subject completed an action sequence than when the second stage action was not executed as part of a sequence (>437 ms). Importantly, this effect was not significant when the previous trial was not rewarded, which rules out the possibility that the observed increase in the reaction times was because of the cost of switching to the other second stage action. Only when (1) the previous trial was rewarded, (2) the subject took the same first stage action, and (3) their reaction time was low did the subject repeat the second stage action, consistent with the prediction of the collaborative hierarchical account.

### Rodent

A number of reports have now been published evaluating two-stage discrimination learning in rodents (Akam et al., 2015; Miller et al., 2017; Groman et al., 2019). In a recent study, using a task modeled on that described for use in humans above, we sought to investigate how the state-space and action representations adapt to the structure of the world during the course of learning without any explicit instructions about the structure of the task – which obviously cannot be provided to rats (Dezfouli and Balleine, 2019) – see Figure 2E. Briefly, we found evidence that, early in training, the rats made decisions based on the assumption that the state-space was simple and the environment composed of a single stage, whereas, later in training, they learned the true multistage structure of the environment and made decisions accordingly – Figures 2F,G. Importantly, we were also able to show that concurrently with the expansion of the state-space, the set of actions also expanded and action sequences were added to the set of actions that the rats executed in similar fashion to human subjects – Figure 2D vs. Figure 2H: human vs. rat data.

In more detail, the lack of instructions implies that the rats have first to establish the nature of what might be called the “task space,” in this case, the fact that the task has two stages. This means that the rats needed to use feedback from the previous trial to track which stage 2 state was rewarded so as to take the stage 1 action leading to that state. It was clear that, early in training, the rats responded as if the first stage was not related to the second stage; as shown in Figure 2F, the rats failed to show a tendency to take the same stage 1 action after earning a reward on the previous trial and instead tended to repeat the action taken immediately prior to reward delivery; i.e., if they took “L” at stage 1, and “R” at stage 2 and earned reward, then they repeated action “R” at the beginning of the next trial. Therefore, actions were not based on a two-stage representation. Importantly, however, this pattern of choices reversed as the training progressed and the rats started to take the same stage 1 action that earned reward on the previous trial rather than repeating the action most proximal to reward – Figure 2F. Clearly, the rats had learned that the task has two stages and, at that point, acquired the correct state-space of the task. If this is true, however, then, during the course of training, the task space used by the animals expanded from a simple representation to a more complex representation consistent with its two-stage structure.

Importantly, learning the interaction of the two stages of the task is not the only way that the rats could have adapted to the two-stage structure of the environment; as mentioned above, in this task, reward can be earned either by executing simple actions in each stage or an action sequence; i.e., the rats could have learned to press the left or the right lever in series and/or to perform left → right or right → left as a chunked sequence of actions. Using these expanded actions, the rats could then repeat a rewarded sequence instead of merely repeating the action proximal to the reward. If this is true, however, then the transition in the pattern of stage 1 actions shown in Figure 2F could have been due to the development of action sequences rather than learning the task space. To establish whether the rats were using chunked sequences of actions, we examined their choices in probe test sessions in which the common (trained) transitions from stage 1 were interleaved with rare transitions; meaning that, after repeating the same stage 1 action, rats could end up in a different stage 2 state than on the previous trial – see Figure 2G for 1st stage choices and Figure 2H for 2nd stage choices. In this situation, we should expect them to take a different stage 2 action, if they were selecting actions singly, whereas, if they are repeating the previously rewarded sequence, they should take the same state 2 action. In fact, the data revealed clear evidence for the latter and for the fact that the rats were using action sequences in this way – Figure 2H. Generally, if the previous trial was rewarded and the rats stayed on the same stage 1 action, then they also tended to repeat the same stage 2 action. Therefore, the pattern of choices at stage 2 we observed was consistent with the suggestion that the rats expanded the initial set of actions to a more complex set that included action sequences.

Hence, exactly as we found in human subjects, we found evidence that rats could incorporate both simple actions and complex action sequences into their repertoire and that, when responding on a sequence, the actions in the sequence were performed regardless of their specific consequences. We also sought to establish the computational model that best characterized the decision-making process used by the rats comparing non-hierarchical model-based RL, hierarchical model-based RL, and a hybrid model-based RL and model-free RL and found, using Bayes model comparison, that hierarchical model-based RL provided the best explanation of the data.

Taken together, these experiments provide consistent evidence, across species in rats and humans, that a hierarchical collaborative process mediates instrumental performance in which simple actions and chunked sequences of actions are available for evaluation by the same goal-directed control process in associative memory and, when positively evaluated, add similarly to the impetus for those urges to be executed.

## Discussion

The issue of how to identify a habit is rapidly becoming an important one for neuroscience and behavioral analyses of decision-making and action control to resolve. The suggestion that habits are merely the obverse of goal-directed actions, i.e., are actions that can be shown to be insensitive to their causal consequences and to the value of those consequences, is simply too broad. Many actions will appear habitual by these criteria when they are not, and, as mentioned above, in practice, these criteria devolve to asserting the significance of the null hypothesis.

### Defining Habits

In order to overcome this issue, positive qualities of habitual control need to be specified. Within the current framework, we advanced the claim that one way to identify habits is *via* their relationship to other actions within chunked action sequences. Habits, it was claimed, are not single solutions but sit within a flow of stimuli and responses with internal response-induced stimuli supporting the initiation of each subsequent action in a sequence of actions. This is not to say that sequences of this sort cannot be quite short, even though, with continuing practice, they are likely to become quite elaborate. Rather it is claimed that any action that is habitual will be performed in an open loop manner; that its antecedent causes are the effects of the immediately preceding action and its consequences relevant only for the next response in the chain. From this perspective flows other potential features of habits; for example, their chronometry: the reduced reaction time, and increased speed of movement that accompanies these kinds of action spring immediately from the nature of action sequences as open loop systems. The lack of dependency of each sequential movement on feedback from their external consequences ensures that each movement can be initiated quickly. Similarly, the refinement of each movement through repetition and its association with its specific eliciting conditions within the sequence ensures its topographical similarity across instances (meaning the invariance in the kinematics of the motor movement). Habits, then, are actions shown to accord with four distinct observations: (1) relatively rapidly deployed and executed, (2) relatively invariant in topography, (3) incorporated into chunked action sequences, and (4) insensitive to changes in their relationship to their individual consequences and the value of those consequences.

### Actions and Habits Do Not Compete

The division of actions and habits into separate and competing control processes is difficult to sustain when their level of collaboration is fully recognized. As described here, the evidence points strongly to the integration of S-R and R-O selection processes through which the various options for action are evaluated. An urge can then be acted upon, whether through a single response or a sequence of responses, or it can be withheld. In some cases, the strength and speed of an urge can produce slips of action; i.e., actions that would otherwise have been withheld. In others, the selection of an action that is part of, or similar to an action that is part of, a sequence can result in “action capture” and the unintentional completion of a sequence of responses inappropriate to the situation. These errors are anticipated from a hierarchical control perspective, whereas from a competitive perspective they are not.

Although the behavioral, neural, and computational evidence for competition between controllers seems overwhelming, careful consideration of this evidence suggests that much of it is open to reinterpretation. From the current perspective, for example, the general claim is that factors argued to influence arbitration between goal-directed and habitual controls can be as readily argued to influence choice between simple actions and action sequences. Costs and benefits influencing this selection process will do so for much the same reason that has been suggested previously; except, of course, the emphasis will be largely on the reduced cost associated with selecting sequences and the potentially increased rewards associated with simple actions due to their more immediate adjustment to environmental constraints based on feedback. Similarly, to the extent that cognitive load and increased planning complexity favor habits (see, for example, Otto et al., 2013), a model-based controller should be expected to select action sequences more than simple actions. This is because the evaluation of action sequences is less cognitively demanding than a set of single actions as the former do not rely on calculating the value of middle states. Similarly, with changing planning complexity; in simple environments planning can be handled by individual actions, which have a higher accuracy, but as the environment becomes more complex the reliance on action sequences becomes more important because the cost of evaluating individual actions increases exponentially with the complexity of the environment. Nevertheless, although many of the interpretations of the behavioral and neural evidence have generated definitions of habit that are, ultimately, circular, the computational approach is different in this regard. The evidence from tasks and models is impressively closely related. Much of this evidence has, however, been driven by a number of simplifying assumptions that in many ways beg the question; such as equating habits with reward-related repetition and so with model-free control.

### Computational Collaboration

We contend, therefore, that an architecture favoring the collaboration between controllers makes greater sense of the data, appears less subject to arbitrary assumption, and so more open to test. We advanced these ideas here by relating a hierarchical reinforcement learning approach to the functions of the associative memory in an associative-cybernetic model of instrumental conditioning. The mechanics of the individual actions, or action sequences, we assume to be the province of the S-R memory, and the evaluation of these actions, including their costs, to be determined by an incentive memory. This provides a simple “algorithmic level” architecture within which collaboration is structurally determined through the selection-evaluation-execution of simple actions or action sequences and is amenable in computational terms to hierarchical reinforcement learning.

Perhaps for this reason, several computational accounts appear, superficially at least, to have similar features to the hierarchical account. For example, one collaborative view, Dyna (Sutton, 1991; see also Gershman et al., 2014; Momennejad et al., 2018), proposes that model-based replay can train the model-free system; a suggestion that devolves to something like rehearsal or perhaps consolidation. An animal simulating or thinking through previous choices through the steps of a decision tree could provide sufficient instances to enable a model-free system to learn more rapidly. This is, however, clearly *learning*-related collaboration; goal-directed and habitual controllers are collaborating in training habitual actions, not in the *performance* of instrumental actions generally. Although one could certainly imagine this kind of process contributing to the consolidation or chunking of habitual sequences of actions, it is not clear how it would function to select between the various options subsequently. It could, as has been argued (Momennejad et al., 2018), improve goal-directed planning, but in that case it remains unclear whether such improvement is due to better integration of performance factors or improved encoding of task structure.

Another interesting example is that of Cushman and Morris’ (2015) habitual goal selection theory, which inverts the relationships described here, proposing model-free control over hierarchical goal selection. From this perspective, a habit controller provides the animal with goals toward which it can plan in a goal-directed manner. These ideas are interesting but require significant broadening of what is traditionally taken to be the subject matter of habitual control. More typically in the literature the goal of a habit is taken to be a specified motor movement; it is not a state of affairs in the world. An animal working to change the world to accord with its desires is usually taken to be working in a goal-directed manner; its aim is an external goal-state and the way in which its actions achieve that state is of only secondary importance (e.g., whether the rat presses the lever with its paw or its elbow is immaterial to ensuring delivery of a food pellet). In many ways, Cushman and Morris’ claims have much in common with theories emphasizing the function of discriminative cues, such as occasion-setters in hierarchical S-(R-O) theories of instrumental action (Rescorla, 1991). On such views these associations are modulatory; the stimulus modulates the selection and performance of specific actions in a hierarchical fashion and not as a S-R habit. Within the hierarchical-cybernetic model described here, Cushman and Morris’ habitual controller would not lie in the habit memory but would modulate action selection in the associative memory in line with associative accounts of modulation. Given the division we have drawn between sequential and simple goal-directed actions, therefore, we suggest that habitual goal selection theory applies more directly to goal selection within the goal-directed system and is not related to habits.

An explicitly performance-based collaborative account has also been developed by Keramati et al. (2016) based on a “planning until habit” approach; i.e., a certain amount of goal-directed planning is undertaken until a habit is selected at which point the habit takes over the control of performance. This account has potentially a great deal more in common with the hierarchical approach because habits are nested within the goal-directed planner which selects habits at some point in the decision tree to complete the action; essentially a model-based process uses model-free values at the end of the decision tree to complete the action. In contrast, the hierarchical approach to habit described above can be implemented using hierarchical RL which eschews a description of this process as model-free. A similar approach is taken in a recent paper by Miller et al. (2019) who argue that habits are mediated by a value-free perseverative process that, following Thorndike’s law of exercise and Guthrie’s contiguity account, is determined by repetition alone. From this perspective, goal-directed actions are mediated by model-free and model-based processes, the former when outcomes are represented by their general affective qualities and the latter when they are characterized by their specific sensory properties. Nevertheless, these forms of action control do not collaborate and their interaction remains both competitive and mediated by an arbitrator, the latter sensitive to the strength of the action-outcome contingency.

It may be possible within a value-free model of habits to develop an account of chunked action sequences in which they are mediated by motor stimuli, much as we have argued for the integrated hierarchical-cybernetic model above. However, from the value-free perspective, if such sequences are habitual they will also be value-free and there is good evidence to suggest that this is not the case. For example, Ostlund et al. (2009) trained rats on two action sequences and found that, although the individual responses of which they were composed were insensitive to outcome devaluation and contingency degradation, these manipulations reduced the performance of the specific sequences that delivered the devalued or the non-contiguous outcome during these tests. Thus, although the individual actions in the sequences appeared habitual, the sequences themselves were clearly goal-directed.

## Author Contributions

BB wrote the paper. AD edited the paper.

### Conflict of Interest

The authors declare that the research was conducted in the absence of any commercial or financial relationships that could be construed as a potential conflict of interest.
